# Renal cell carcinoma treated with stereotactic radiotherapy with histological change confirmed on autopsy: a case report

**DOI:** 10.1186/1756-0500-7-270

**Published:** 2014-04-26

**Authors:** Hiroshi Onishi, Tomonori Kawasaki, Hidenori Zakoji, Takashi Yoshida, Takafumi Komiyama, Kengo Kuriyama, Masayuki Araya, Ryo Saito, Shinichi Aoki, Yoshiyasu Maehata, Licht Tominaga, Kan Marino, Iori Watanabe, Mitsuhiko Oguri, Tsutomu Araki, Nobuyuki Enomoto, Masayuki Takeda, Ryohei Katoh

**Affiliations:** 1 Department of Radiation Oncology, University of Yamanashi, 1110 Shimokato, 409-3898 Chuo-city, Yamanashi, Japan; 2 Department of Human Pathology, University of Yamanashi, 1110 Shimokato, 409-3898 Chuo-city, Yamanashi, Japan; 3 Department of Urology, University of Yamanashi, 1110 Shimokato, 409-3898 Chuo-city, Yamanashi, Japan; 4 First Department of Internal Medicine, University of Yamanashi, 1110 Shimokato, 409-3898 Chuo-city, Yamanashi, Japan

**Keywords:** Renal cell carcinoma, Stereotactic body radiation therapy, Histological effect, Autopsy

## Abstract

**Background:**

Treatment of primary renal cell carcinoma using radiotherapy with curative intent is rare, because renal cell carcinoma is generally regarded as a radiation-resistant tumor. Recently, stereotactic body radiation therapy has been radically applied for cancers in various organs including renal cell carcinoma. However, there were few reports describing pathological changes of renal cell carcinoma post stereotactic body radiation therapy. This is the first report we are aware of documenting late histological effects of stereotactic body radiation therapy on renal cell carcinoma and surrounding normal tissue.

**Case presentation:**

A right renal tumor was identified in a Japanese 70-year-old man on follow-up computed tomography for his chronic hepatitis. T1N0M0 renal cell carcinoma was clinically diagnosed as the tumor was 3 cm in diameter and well-enhanced with intravenously infused contrast material in the arterial phase on computed tomography. No metastases in regional lymph nodes or distant sites were evident. Stereotactic body radiation therapy was selected as an alternative therapy to surgery because of his poor liver function. A total dose of 60 Gy in 10 fractions over 12 days was delivered using a 10-megavolt X-ray. The renal tumor gradually decreased in size and partial response had been achieved at 2 years after completing stereotactic body radiation therapy. Hepatocellular carcinoma was identified during follow-up in the patient and he died of progression of hepatocellular carcinoma with hepatic failure 2.5 years after completing stereotactic body radiation therapy. Autopsy was done and it showed almost complete necrosis of tumor tissues with a small amount of viable renal carcinoma cells. These pathological findings suggested marked effects of stereotactic body radiation therapy on clear cell renal cell carcinoma.

**Conclusion:**

Our case demonstrates a good pathological response with small foci of remnant viable cancer cells after stereotactic body radiation therapy of 60Gy in 10 fractions for small renal cell carcinoma. Although further experiences and longer follow-up are mandatory to conclude the optimal treatment schedule and efficacy of stereotactic body radiation therapy for renal cell carcinoma, stereotactic body radiation therapy may represent a novel less-invasive option for the treatment of primary renal cell carcinoma.

## Background

In the recent era of rapidly aging populations, minimally invasive treatment modalities are preferred for elderly patients with various cancers, including renal cell carcinoma (RCC). RCC accounts for approximately 2% of all new cancer incidences worldwide, and has traditionally thought to be a radio-resistant malignancy [1,2]. Radiotherapy (RT) has thus played little role in the management of primary RCC, and few reports have described curative radiotherapy for primary RCC. Conventional radiation to a total of 60 Gy or so in 1.8- to 2-Gy daily fractions has not proven very effective in controlling the tumor in curative or adjuvant settings. The role of conventional RT is mainly palliative for metastatic disease, producing a subjective or objective response in about 50% of symptomatic patients [2]. Brain metastases from RCC have recently been successfully treated with stereotactic radiosurgery (SRS), providing local control rates exceeding 85% [3,4]. Advances in technology and methods in radiation oncology have led to the clinical implementation of image-guided RT (IGRT) and body stereotaxis, allowing the delivery of very high, biologically potent doses to extracranial tumors. Primary RCC and its metastases to extra-cranial sites may thus now be treated with similar success using stereotactic body radiation therapy (SBRT) [5,6]. SBRT has been available for more than 10 years and is gaining clinical interest as a means of achieving local radical treatment of tumors in various organs, particularly for patients with stage I non-small-cell lung cancer (NSCLC) [7,8], however, histological late effects of SBRT for primary RCC have not previously been shown. We report herein the case of a patient who underwent treatment of primary RCC treated with SBRT and autopsy after his death. This is the first reported case to demonstrate late histological effects on primary RCC and surrounding normal tissue after SBRT.

## Case presentation

**Figure 1:**
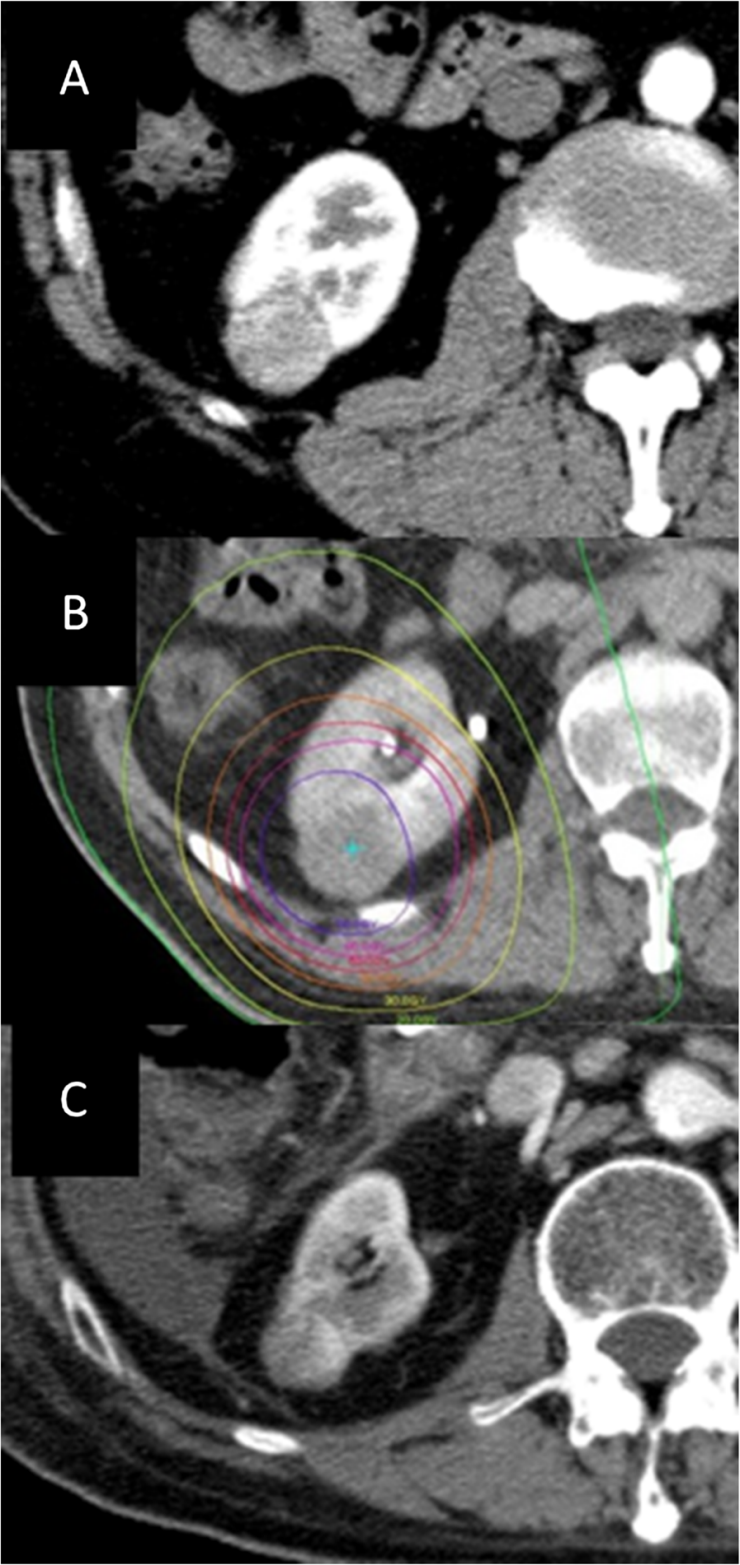
**Initial CT, radiotherapy planning, and follow-up CT. (A)** Axial contrast-enhanced computed tomography image before stereotactic body radiation therapy. **(B)** Dose distribution of the planning for stereotactic body radiation therapy. Isodose lines show 70Gy, 60Gy, 50Gy, 40Gy, 30Gy, 20Gy, and 10Gy from the inside. **(C)** Axial contrast-enhanced computed tomography image two years after SBRT. The renal tumor decreased in size and its surrounding renal portion got atrophic.

On follow-up computed tomography (CT) for chronic hepatitis, a right renal tumor was identified in a Japanese 70-year-old man. The renal tumor was 3 cm in diameter and located in the posterolateral portion of the right kidney (Figure 1A). The lesion showed good enhancement with intravenously infused contrast material in the arterial phase on CT and was considered to be RCC clinically. No metastases in regional lymph nodes or distant sites were evident on whole-body CT, therefore T1N0M0 RCC was diagnosed. The patient was judged as high-risk for surgery due to his poor liver function. He expressed a desire to undergo SBRT as local intensive therapy though we explained to him that SBRT was a new, non-standard treatment for renal cell cancer. To minimize respiratory motion of the kidney during irradiation, the patient was trained in voluntary self-breath-holding during the inspiration phase using a respiratory indicator. Planning target volume (PTV) was determined as the gross tumor volume of the right renal mass plus the personal internal margin with an additional margin of 3 mm to compensate for intra-session reproducibility and to provide a safety margin. Tumor position was adjusted to the planned position before every session using CT images taken in the vicinity of the tumor. Ten different non-coplanar static beams were used for irradiation. The radiation port was made with sliding multileaves adjusted with 3-mm margins around the border of the PTV. A total dose to the center of 60 Gy in 10 fractions over 12 days was delivered using a 10-MV X-ray. Targets delineations and isovalue lines written on CT are shown in Figure 1. Ninety-five percent of the PTV was within the 95% isodose line to the prescribed dose (Figure 1B). No clinical acute or chronic side effects were recorded during or after treatment, which was well tolerated. The renal tumor slowly but continuously decreased in size and partial response had been achieved at 2 years after completing SBRT, and its surrounding renal portion got atrophic (Figure 1C). Hepatocellular carcinoma was identified during follow-up and the patient died of progression of this malignancy and liver dysfunction 2.5 years after completing SBRT. Autopsy was performed and the right renal tumor treated with SBRT was pathologically examined.

**Figure 2:**
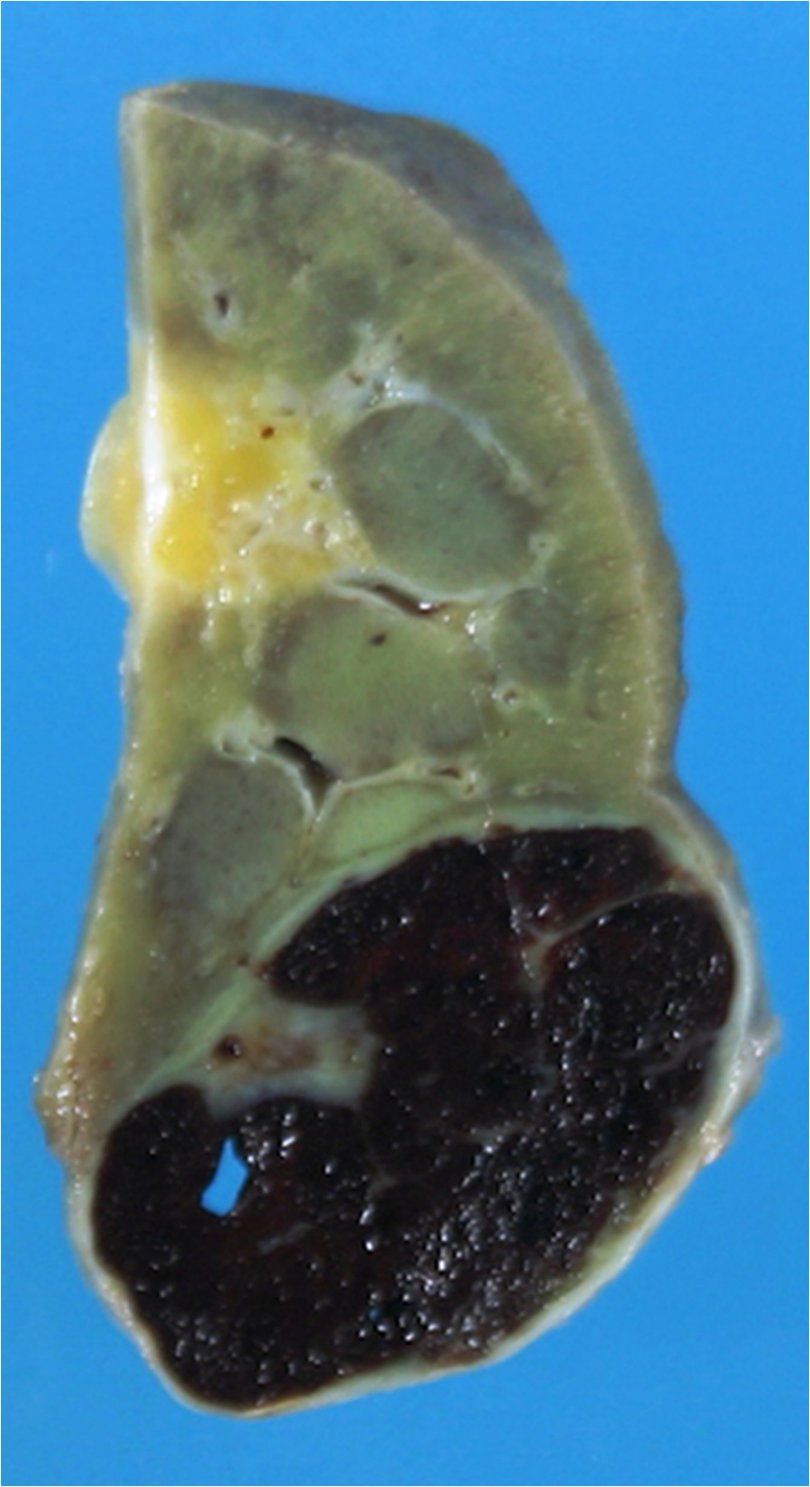
**Macroscopic findings of clear cell renal cell carcinoma treated with stereotactic radiotherapy.** On the cut surface of the autopsied kidney tissue, a 2.4 × 1.6 cm tumor mass with clear margins, blackish-brown in color, is recognizable in the atrophic kidney tissue.

**Figure 3:**
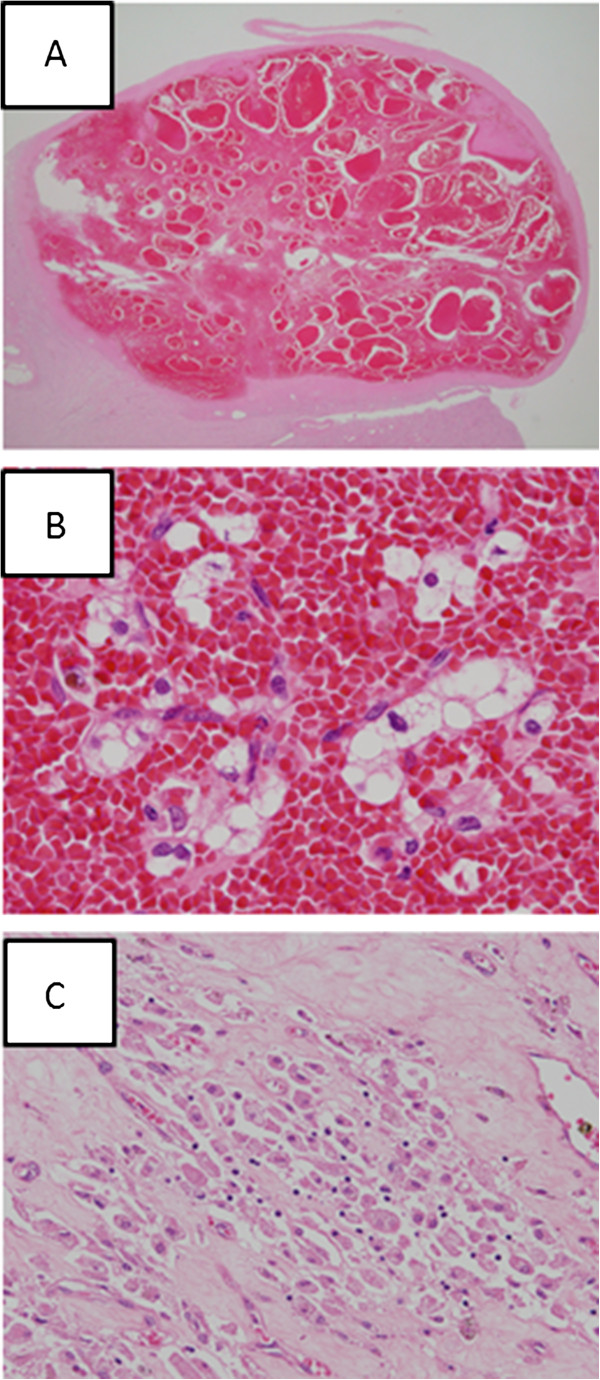
**Histological findings of clear cell renal cell carcinoma treated with stereotactic radiotherapy. (A)** The tumor at low magnification shows markedly hemorrhagic findings within a fibrous capsule. **(B)** A high-power view demonstrating clustered, degenerative cancer cells with abundant, clear and/or vacuolated cytoplasm and round-to-ovoid or irregularly-shaped nuclei with a hemorrhagic background. **(C)** Aggregation of foamy macrophages in the fibrotic stroma.

On autopsy, a well-circumscribed, blackish-brown solid tumor, measuring 2.4 × 2.0 × 1.6 cm, was evident on the cut surface of the lower portion of the right kidney specimen accompanying atrophic change (Figure 2). Histologically, this tumor was surrounded by a distinct fibrous capsule and almost the entire tumor showed hemorrhagic necrosis (Figure 3A), although a small number of degenerative carcinoma cells with abundant, clear and/or vesicular cytoplasm and nuclei with mild to moderate pleomorphism remained (Figure 3B). Focal fibrosis, edema and aggregated foamy macrophages were also observed (Figure 3C). These pathological findings suggested marked effects of SBRT on clear cell RCC.

## Discussion

Conventional radiotherapy is not considered to have a role to play in the definitive management of RCC. SBRT offers the only non-invasive, highly efficient means of eradicating small-sized tumors at primary or metastatic sites. Effects of hypofractionated single high-dose irradiation on RCC have been demonstrated in several papers. In an animal study, Walsh *et al.* irradiated a nude mice xenograft model injected subcutaneously with A498 human renal carcinoma cells with three fractions (one per week) for a total dose of 48 Gy [9]. All irradiated tumors subsequently decreased gradually in size and exhibited marked cytological changes. In a human study, Svedman *et al*. reported a good local control rate of 98% on imaging after SBRT noted in a prospective study involving 30 patients with metastatic or inoperable primary RCC [6]. Gerszten *et al*. showed that two patients with medically inoperable primary RCC also did well with pain relief and stable disease on imaging [10]. Beitler *et al*. described a series of nine patients with primary RCC treated with SBRT, noting four long-term survivors (minimum follow-up, 48 months) [11]. Teh *et al*. concluded that image-guided SBRT provides excellent local control on imaging without any significant toxicity, and SBRT may represent a novel, non-invasive, nephron-sparing option for the treatment of primary RCC [12]. Histologically, Ponsky *et al.* reported three cases of pathological evaluation of RCC at eight weeks after SBRT [13]. In the report, histological complete response was found in one of three patients with primary RCC after receiving SBRT dose of 16 Gy in 4 fractions. To our knowledge, it is the only report describing histological change of primary RCC after SBRT, however the duration between SBRT and histological examination was very short (eight weeks). Histologic effects on the tumor and surrounding normal tissues would be expected to change after eight weeks. In addition, the dose of SBRT in Ponsky’s report is much lower than that of ours. Therefore we believe the current case report has a novel finding that demonstrated late histological effects at 2.5 years after high dose SBRT on primary RCC.

Essentially no significant treatment-related toxicity was noted in patients with metastatic RCC and primary RCC treated with SBRT in above reported series, including the present case. This is likely due to the precise delivery of high-dose radiation and the use of stereotactic and IGRT technology. Because of the rapid falloff in isodose lines, only very limited normal tissues beyond the tumor target received high-dose radiation. No severe deterioration of renal function as well as functions of other normal organs was seen in patients whose primary RCC received SBRT. SBRT may also play an important role especially in patients with recurrent RCC in the remaining kidney, for which the preservation of renal function is of utmost importance. In contrast to other local therapeutic modalities such as radio-frequency ablation, surgery and cryotherapy, radiotherapy is the least invasive therapeutic procedure for patients.

Determining the optimal SBRT fractionation schedule is difficult, because of heterogeneity in the doses used and because the true α/β of RCC is not clear. Ning *et al*. determined α/β values for RCC cell lines A498 and Caki-1 as 2.6 and 6.92, respectively [14]. If the α/β of RCC is very small, the large fraction size of SBRT may be advantageous to decrease chronic adverse effects on surrounding normal tissue while maintaining a biological effect for tumor control. More work is required to determine the most appropriate α/β values and optimal dose for RCC, especially using the SBRT approach. On the other hand, if hypoxia is one of the important factors determining “radioresistance” in RCC, longer fractionation schedules with moderately high-dose fractions beyond 5–6 fractions may be better than 1–3 fractions with ultra-high dose. According to the local histopathological effects seen in this case, slightly greater tumoricidal efficacy might be necessary. Despite all the above-mentioned shortcomings, the present case showed encouraging pathological effects, but small foci of remnant viable cancer cells after SBRT. Carbon ion radiation therapy may be valuable in order to achieve more strong radiobiological effect and keep normal organs around the kidney away from radiation toxicity [15], but it needs extremely large cost and space.

Although further experience and longer follow-up are mandatory to confirm the optimal treatment schedule and efficacy of SBRT for RCC, SBRT may represent a novel non-invasive, nephron-sparing option for the treatment of primary RCC.

## Conclusion

The present case showed a good pathological response with small foci of remnant viable cancer cells after SBRT of 60Gy in 10 fractions for small RCC. Although further experiences and longer follow-up are mandatory to conclude the optimal treatment schedule and efficacy of SBRT for RCC, SBRT may represent a novel non-invasive, nephron-sparing option for the treatment of primary RCC.

## Consent

Written informed consent was obtained from the patient for publication in this case report and any accompanying images. A copy of the written consent is available for review by the Editor-in-Chief of this journal.

## Abbreviations

SBRT: Stereotactic body radiotherapy; RCC: Renal cell carcinoma; RT: Radiotherapy; IGRT: Image-guided radiotherapy; CT: Computed tomography; PTV: Planning target volume.

## Competing interests

The authors declare that they have no competing interests.

## Authors’ contributions

HO, HZ, TY, TK, KK, KA, RS, SA, YM, LT, KM, IW, MO: provided medical assistance to the patients and defined the treatment approach; HO, KM, TK, KK: contributed in the treatment planning; TK, RK: provided pathological specimens; HO, TK: contributed in the acquisition, analysis, interpretation of data; HO: drafted the manuscript; TA, NE, MT, RK: gave final revision and approval. All authors read and approved the final manuscript.
